# Advancing surgical setting: A paradigm for healthcare workers during the monkeypox outbreak

**DOI:** 10.1016/j.amsu.2022.104343

**Published:** 2022-08-08

**Authors:** Mohammad Mobin Teymouri Athar, Nastaran Sarvipour, Arman Shafiee

**Affiliations:** School of Medicine, Shahid Beheshti University of Medical Sciences, Tehran, Iran; Faculty of Medicine, Kerman University of Medical Sciences, Kerman, Iran; Clinical Research Development Unit, Alborz University of Medical Sciences, Karaj, Iran; Student Research Committee, School of Medicine, Alborz University of Medical Sciences, Karaj, Iran

To the Editor,

Monkeypox is a rare zoonotic disease caused by the monkeypox virus (MPV), an enveloped double-stranded DNA virus that belongs to the Orthopoxvirus genus of the Poxviridae family. It was first discovered as an outbreak in a cluster of monkeys in a Danish research institute in 1958. The first case in a human was a 9-month-old child recognized in 1970 in the Democratic Republic of the Congo, DRC [1]. Since then, the virus has been circulating mainly in Central and West Africa; however, sporadic outbreaks outside Africa have been reported over the last two decades. These occasional outbreaks were all related to travel to Africa except the 2003 outbreak in the USA, whose origin was the imported rodents infected with the virus [2]. In the current outbreak starting in the UK in May 2022, more than 7000 monkeypox cases were reported from 57 non-endemic countries, causing global concern [3]. Two clades have been identified, the West African and Central African, named after their geographical distribution. Both clades cause similar clinical syndromes with the Central African being associated with more severe forms of the disease, leading to a three-fold mortality rate in comparison with the West African variant, the one currently circulating outside Africa [4]. Clinical manifestations of the disease include a febrile prodrome with headache, lymphadenopathy (submandibular, cervical, postauricular, axillary, or inguinal), muscle aches, and malaise. This prodrome is followed by vesicular or pustular skin rashes with centrifugal distribution, mostly occurring on the face, palms of the hands, soles of the feet, mucosal membranes, genitalia, and conjunctiva. The disease is generally self-limited; however, children, pregnant women, and immunocompromised patients are at risk of severe disease and further complications [5,6]. The virus is transmitted either through animal-human or human-human route. Human-human transmission is possible via large respiratory droplets and contact with body fluids, contaminated surfaces of objects surrounding the patients such as clothing and linen, and skin lesions of an infected person. Reports of nosocomial transmission have also been found [7,8]. Prolonged contact with patients places healthcare providers and family members at risk of infection [9]. Therefore, it is crucial to acquire proper preventive measures to hinder the transmission of the virus through the aforementioned routes.

Poxviruses are very stable and remain contagious for long periods of time. A study conducted in Germany provided evidence of surface contamination in hospital rooms, stating the importance of protection measures against the monkeypox virus. Healthcare providers must apply personal protective equipment (PPE) while regularly disinfecting the frequent contact spots on the body surface, double gloving, and proper hygiene of the hands, in addition to disinfection of the possibly contaminated surfaces [10]. Avoiding direct contact with skin lesions or objects used by the patient, such as clothes, bedding, and towels is necessary for decreasing the risk of infection. Healthcare staff providing care for monkeypox patients with skin lesions should utilize proper PPE including disposable gown and hand gloves, eye protection such as face shields or goggles, and a fit-tested filtering respirator (for example, an N95 mask). Activities that resuspend the virus particles such as shaking bed linens, sweeping, and dry dusting should be avoided. Suspected or confirmed patients should be instantly isolated in separate single-person rooms while being masked and their lesions being covered with a gown or sheet to avoid possible spread of the virus [11,12]. When considering the impact of public health emergencies on the practice of medicine, surgical care is not an exception. We have learned from our experience during the COVID-19 pandemic that the spread of infectious diseases affects the operating volumes and surgical outcomes. Moreover, patients who have undergone surgical operations are more susceptible to severe forms of monkeypox disease. On the other hand, interruptions of surgical care provision are possible due to social distancing and reluctance of patients to admit to hospitals for surgical procedures, as well as the increased infection rate among healthcare staff and patients. These prolong waiting times and negatively affect the outcomes in terms of morbidity and mortality [13]. Discontinuation of elective surgery based solely on symptoms and diagnostic tests, without considering the patients’ conditions and complications due to delayed surgery, has had a major impact on the treatment process for patients around the world [14].

Preventive measures in the setting of surgery in the time of COVID-19 as a recent experience of public health emergency could be employed in the context of monkeypox disease (Fig. 1). Strict hospital regulations, including daily temperature screening, a dedicated operating room for both confirmed or presumptive patients, and postponing elective surgeries, along with the utilisation of risk assessment tools to prioritise patients based on the urgency of their operation, decrease the risk of transmission [15]. The surgical team should strictly follow the guidelines designed for infection control and disease prevention including the use of PPE to prevent direct contact or exposure to aerosols formed during the surgical procedures [16].

**Fig. 1:**
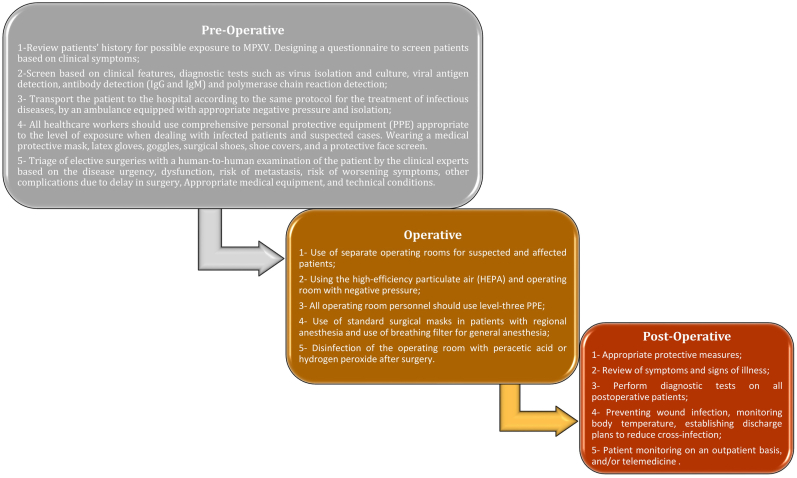
Developed diagram for strategies that should be considered during the outbreak of Monkeypox in the surgical setting. The paradigm was classified into 3 main categories: preoperative assessment, operating room assessment, and postoperative management.

In addition to potential aerosol-generating procedures (AGPs) such as cardiopulmonary resuscitation, surgical smoke, suctioning, intubation, and extubation, the techniques and tools used, such as electric cautery, pulse washing, and laparoscopic suction devices in transoral robotic surgery (TORS), minimally invasive surgery (MIS), laparoscopic and video-assisted thoracoscopic surgery (VATS) procedures, and other surgeries also generate aerosols infected with the virus [17,18].

Among the measures that can be taken in the field of anesthesia to reduce aerosol production include the use of rapid sequence intubation (RSI), face mask ventilation, open airway suction, prioritization of using regional anesthesia if possible, and use of positive pressure ventilation [19,20].

Decreasing application time, improving tissue moisture to reduce surgical smoke, limitation of the use of equipment and procedures such as harmonic scalpels, use of smoke evacuation system and filtration of CO_2_ plume, creation of a water-tight, sterile and isolated environment with sterile plastic drapes help to reduce the transmission of monkeypox and the formation of aerosol particles [18,21]. Restriction of the operating team to the most experienced staff with the minimal number of members leads to minimization of operating time, blood loss, duration of exposure, morbidity, and length of hospital stay. Altogether, these precautions will lower the chances of viral transmission, thus protecting other patients and healthcare providers [22]. In conclusion, with applying our knowledge obtained from the COVID-19 pandemic in terms of disease control and prevention to the recent outbreak of monkeypox, it is possible to overcome the future challenges encountered in healthcare facilities, particularly the setting of surgery, regarding infection control.

## Ethical approval

Not applicable.

## Source of funding

No funding was received.

## Author contribution

Mohammad Mobin Teymouri Athar, Nastaran Sarvi-Pour: Conceptualization, Investigation, Writing- original draft. Arman Shafiee: Conceptualization, Investigation, Project administration, Writing-review & editing.

## Trail registry number

Not applicable.

## Guarantor

Arman Shafiee.

## Provenance and peer review

Not commissioned, externally peer reviewed.

## Data availability statement

Data sharing is available by contacting corresponding author.

## Declaration of competing interest

Not applicable.
